# Endometrial gene expression profiling in pregnant Meishan and Yorkshire pigs on day 12 of gestation

**DOI:** 10.1186/1471-2164-15-156

**Published:** 2014-02-24

**Authors:** Ting Gu, Meng-jin Zhu, Martine Schroyen, Long Qu, Dan Nettleton, Dan Kuhar, Joan K Lunney, Jason W Ross, Shu-hong Zhao, Christopher K Tuggle

**Affiliations:** 1 Key Laboratory of Agricultural Animal Genetics, Breeding and Reproduction of Ministry of Education, Huazhong Agricultural University, Wuhan 430070, P. R. China; 2 Department of Animal Science, Iowa State University, Ames 50011, USA; 3 Department of Statistics, Iowa State University, Ames 50011, USA; 4 Animal Parasitic Diseases Laboratory, BARC, ARS, USDA, Beltsville, MD 20705, USA

**Keywords:** Pig, Prolificacy, Endometrium, Differentially expressed genes, Implantation

## Abstract

**Background:**

Litter size in pigs is a major factor affecting the profitability in the pig industry. The peri-implantation window in pigs is characterized by the coordinated interactions between the maternal uterine endometrium and the rapidly elongating conceptuses and represents a period of time during which a large percentage of the developing conceptuses are lost. However, the gene expression and regulatory networks in the endometrium contributing to the establishment of the maternal: placental interface remain poorly understood.

**Results:**

We characterized the endometrial gene expression profile during the peri-implantation stage of development by comparing two breeds that demonstrate very different reproductive efficiencies. We employed the porcine Affymetrix GeneChip® to assay the transcriptomic profiles of genes expressed in the uterine endometrium obtained from Meishan and Yorkshire gilts (n = 4 for each breed) on day 12 of gestation (M12 and Y12, respectively). Total of 17,076 probesets were identified as "present" in at least two arrays. A mixed model-based statistical analysis predicted a total of 2,656 (q < 0.1) transcripts as differentially expressed between Meishan and Yorkshire pigs. Eighteen differentially expressed transcripts of interest were validated by quantitative real-time PCR. Gene ontology (GO) annotation revealed that the known functions of the differentially expressed genes were involved in a series of important biological processes relevant to early pregnancy establishment in the pig.

**Conclusions:**

The results identified endometrial gene expression profiles of two breeds differing in litter size and identified candidate genes that are related to known physiological pathways related to reproductive prolificacy. These findings provide a deeper understanding of molecular pathways differing between two breeds at the critical peri-implantation stage of pregnancy, which can be utilized to better understand the events contributing to pregnancy establishment in the pig.

## Background

The pig (*Susscrofa*) has been used as model animal in many fields, including biological [1], agricultural, and biomedical studies [2-4]. Pig litter size, which can be impacted by multiple factors including ovulation rate, embryonic survival, uterine capacity and fetal survival, is one of the most important economic traits affecting production efficiency in the pig industry [5]. Improvements in litter size across the swine industry in recent decades has occurred through different selection schemes such as phenotypic selection, family index selection, BLUP-based selection, component trait selection, and hyperprolific selection [5,6]. However, despite current improvements, the low heritability of litter size has limited the genetic selection progress in substantially improving this trait [7]. Improvement of litter size is ideally suited for application of molecular breeding approaches such as marker-assisted selection (MAS) and whole-genome selection, where molecular markers associated with superior trait values may accelerate genetic improvement. In addition, numerous studies have attempted to detect candidate genes underlying the molecular mechanism of sow prolificacy. Although some candidate genes for sow prolificacy have been reported [8], true causal genes remain scant. A deeper understanding of the physiological and molecular mechanisms associated with reproductive efficiency is needed to further the biological underpinnings that contribute to controlling litter size in pigs.

It is now well established that prenatal mortality can be a major determinant of litter size in pigs [5,9,10]. When early conceptuses undergo rapid differentiation and expansion of their trophoblastic membranes during the first few weeks of gestation, abnormalities of embryogenesis that produce embryonic mortality can occur [5]. The extent and timing of embryonic and fetal losses during gestation has been well documented by previous studies, and it is estimated that about 25-45% of fertilized ova do not survive through gestation [9]. The majority of embryonic loss has been demonstrated to occur before day 20 of gestation, primarily around day 11–12 [10]. Early events in conceptus development, including rapid trophoblastic elongation and successful establishment of the fetal: maternal interface, play a key part in determining litter size. The conceptus itself undergoes a dramatic transcriptomic reorganization during trophoblastic elongation and during initial interactions with the uterine endometrium [11]. Additionally, the copious amounts of estrogen synthesized and released by the elongating conceptus serves as the maternal recognition of pregnancy signal around day 12 of gestation [12]. The timing of endometrial exposure to estrogen is critical as specific molecular cues associated with uterine receptivity occur simultaneously with conceptus estrogen synthesis and abnormal exposure to estrogen prior to day 12 results in compromised pregnancy establishment [13-15].

Studies have revealed that there are very different patterns of gene expression in uterine endometrium between successful and abortive embryo implantation, as well as between high and low litter size breeds [16,17]. It is now clear that the molecular events happening in the maternal endometrium during the early stages play a crucial role in determining the success of embryonic survival, and the early-stage endometrium provides an ideal window to investigate the molecular mechanisms that may be associated early conceptus loss and sow reproductive prolificacy. Many genes involved in angiogenesis and immune responses are expressed at the endometrium-fetal surface [18]. Interestingly, polymorphisms in genes expressed during embryonic implantation, such as in EphA4 and TGF-β, have been associated with litter size [19,20]. However, what is lacking is a systematic comparison investigating global gene expression patterns in the uterine endometrium during the peri-implantation stage of pregnancy between pig breeds with established differences in litter size.

Microarrays have been widely used to detect differentially expressed genes hypothesized to contribute to controlling litter size. Some interesting genes, such as steroidogenic acute regulatory protein and interleukin-1beta (IL1B), were found to be expressed in during early development using multiple global expression profiling approaches [21-24]. Differentially expressed genes have been detected in endometrium between pregnant and non-pregnant sows [25] and between endometrium of normal and developmentally delayed fetuses [26]. Furthermore, genes involved in several important pathways regulating fetal development have been identified comparing gestational day 75 and 90 placentae from Erhualian and Large White pig breeds [27].

Differences in reproductive efficiency exist between different pig breeds. The prolific Meishan pigs have higher embryo survival rates during early gestation,which may be due to the fact that the Meishan conceptus exhibits a reduced trophectoderm mitotic rate during the preimplantation period, elongates from fewer cells and remains smaller throughout gestation compared with other low litter size breeds [28]. To further the comparison between Meishan and domestic swine females, reciprocal embryo transfers were performed and revealed that litter size is controlled primarily by the recipient females rather than the sire or embryos [29-31]. These data suggest that developing a more comprehensive understanding of the maternal uterine microenvironment influencing embryonic survival and implantation during the establishment of pregnancy could be of significance towards understanding maternal mechanisms contributing to successful pregnancy establishment.

In the present investigation we conducted a genome-wide screen to characterize gene expression of endometrial tissue on day 12 of gestation, during maternal recognition of pregnancy. We used the Affymetrix GeneChip® to analyze endometrial gene expression in Meishan and Yorkshire, which have phylogenetically distinct genomes and also have striking prolificacy differences with respect to litter size. We analyzed these endometrial transcriptomic data through a comparative annotation approach to gain a deeper molecular insight into genes controlling the prolific nature of pigs. A number of genes and transcripts were detected to be differentially expressed in endometrial tissue between breeds. The data and our analysis in this investigation contribute toward a deeper understanding of the molecular events associated with reproductive success during early pregnancy establishment by comparing the molecular events between two separate breeds with different levels of prolificacy.

## Results

### Transcriptome analysis in early pregnancy endometrium

The Affymetrix Porcine GeneChip® was used for transcriptomic analyses of endometrium from Yorkshire and Meishan pigs early in pregnancy with four biological replicates for each pig breed. To compare similar stages of endometrial development during gestation, we collected tissue from pregnant Meishan and Yorkshire gilts bred at their second estrous cycle to boars of the same breed. At the time of endometrial tissue collection, we determined the morphological stage conceptuses present with the uteri. We only processed endometrial tissue for this comparison when all embryos were at the filamentous stage. If the signal of a transcript from at least two of the four biological replicates were called “present” by the Affymetrix algorithm, we considered this transcript expressed in the endometrium. Using this criterion, 14,951 transcripts were identified in Meishan day 12 (M12) endometrium, and 14,558 transcripts were detected in Yorkshire day 12 (Y12) endometrium.

### Identification of differentially expressed genes

Following statistical analysis, we identified 2656 (q < 0.1) transcripts that were differentially expressed between M12 and Y12 (Table 1). Among these transcripts, 2264 were annotated as known genes; thus about 15% of the transcripts were unable to be annotated. Among the differentially expressed genes, 1180 were significantly more abundant in M12 endometrium, while 1084 genes were more abundant in Y12.

**Table 1:** Number of differentially expressed annotated genes between Yorkshire and Meishan endometrium on day 12 of gestation

^**1**^**Comparison**	^**2**^**Fold difference greater than2**	^**3**^**Fold difference greater than 1.5**	^**4**^**Difference based on q value < 0.1**
Meishan > Yorkshire	463 (590)	799 (976)	1180 (1372)
Yorkshire > Meishan	364 (441)	736(884)	1084 (1284)
All	827(1031)	1535 (1860)	2264 (2656)

^1^Transcript abundance differences. Meishan > Yorkshire indicates the comparison identifying transcripts that are more abundantly expressed in Meishan endometrium compared to Yorkshire endometrium on day 12 of pregnancy. Yorkshire > Meishan indicates the comparison identifying transcripts that are more abundantly expressed in Yorkshire endometrium compared to Meishan endometrium on day 12 of pregnancy.

^2^Number of differentially expressed annotated genes with 2-fold or greater difference between breeds. Number in parenthesis indicates total number of differentially expressed transcripts.

^3^Number of differentially expressed annotated genes with 1.5-fold or greater difference between breeds. Number in parenthesis indicates total number of differentially expressed transcripts.

^4^Total number of differentially expressed annotated genes based on a q-value of less than 0.1. Number in parenthesis indicates total number of differentially expressed transcripts.

### Cluster analysis and GO annotations

**Figure 1:**
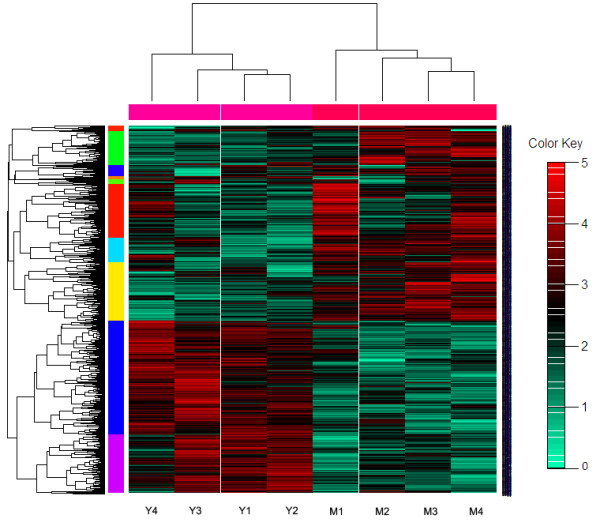
**Hierarchical heat map of differential gene expression as determined by Affymetrix GeneChip® in endometrium of day 12 pregnant Meishan and Yorkshire gilts.** A hierarchical cluster heat map showing the log2 transformed expression values for Affymetrix expression array following hybridization of mRNA prepared from endometrium of Meishan on pregnant day 12 and Yorkshire on pregnant day 12. The individual differentially expressed genes relative expression across the quadruplicates of two breeds is shown horizontally. Green shows lower expression and Red shows higher expression. The genes included are those with q value less than 0.1.

To gain insight into similarities between the transcriptome of the two breeds, data from all the differentially expressed genes in the endometrium were used in a systematic cluster analysis (Figure 1). Although there is substantial variation in expression within the replicates of one breed, the heat map suggests clearly that the selected differential gene set associates the biological samples correctly into two groups each representing one breed. The quality of this association enabled further analysis to identify potential pathways and functions impacted in endometrium using the genes detected at statistically different abundance between these two breeds.

The Database for Annotation, Visualization and Integrated Discovery (DAVID 6.7) was utilized to annotate biological terms and processes preferentially represented in Meishan and Yorkshire endometrium during the peri-implantation stage of development. We used the gene lists of q < 0.1 for DAVID annotation, and tested using the Annotation Chart function in DAVID separately for enrichment of genes with greater abundance in Yorkshire endometrium compared to Meishan endometrium, as well as those with greater abundance in Meishan endometrium compared to Yorkshire endometrium. Terms enriched utilizing these gene lists are provided as Additional file 1: Table S1 and Additional file 2: Table S2.

The five most significant terms in each of the most enriched in the annotation clusters expressed at a higher level in Yorkshire tissue were membrane-enclosed lumen, RNA-splicing, RNA binding, nucleus, and blood vessel development (Table 2). The functions of these enriched genes are closely associated with the nucleus, transcriptional and RNA processing functions in cells. Other functions represented in additional significantly enriched clusters in this list of differentially expressed (DE) genes include apoptosis, cell-cell tight junction, and other functions (Additional file 2: Table S2A). It may be that the Yorkshire endometrium is more transcriptionally responsive to during maternal: fetal crosstalk following exposure to the maternal recognition of pregnancy signals. It is also interesting that genes associated with angiogenesis appear to be more prominently expressed in Yorkshire endometrium. These genes include transcription factors, such as JUN, Foxo1, and angiogenesis genes, such as VEGFC, KDR.

**Table 2:** Most significant gene ontology terms for each of the five most enriched clusters in the gene list representing genes more abundant in Yorkshire endometrium in comparison to Meishan endometrium

**Category**	**Term**^**a**^	**P value**	**Number of associated genes**	**FDR**
GOTERM_CC_FAT	membrane-enclosed lumen	8.83E-10	157	1.28E-6
GOTERM_BP_FAT	RNA splicing	2.86E-6	36	5.15E-3
SP_PIR_KEYWORDS	rna-binding	4.57E-6	53	6.67E-3
SP_PIR_KEYWORDS	nucleus	8.68E-8	283	1.26E-4
GOTERM_BP_FAT	blood vessel development	5.98E-4	27	1.07

^a^DAVID Annotation chart analysis was used to identify the most highly enriched group of related terms (with high enrichment score and small FDR) in the Yorkshire > Meishan list of differentially expressed genes. See entire results in Additional file 2: Table S2A.

Alternatively, the five most significant gene ontology terms enriched in the genes expressed more abundantly in Meishan endometrium were translational elongation, ribosome biogenesis, protein folding, CHORD-containing proteins and SGT1(CS) domain, and intracellular organelle lumen (Table 3). In Meishan endometrium, enriched functions seem to focus on post-transcriptional processes, especially protein synthesis and maturation (Additional file 2: Table S2b). Genes such as ribosome proteins (RPL10, RPL11, RPL12, RPL14, RPL17, RPL18, RPL 35A, RPL36A, RPS2, RPS3, MRPS36) and translation elongation factors (EEF1A1, EEF1A2, EEF1G, EEF2) are more highly expressed in Meishan endometrium.

**Table 3:** Most significant gene ontology terms for each of the five most enriched clusters in the gene list representing genes more abundant in Meishan endometrium in comparison to Yorkshire endometrium

**Category**	**Term**^**a**^	**P value**	**Number of associated genes**	**FDR**
GOTERM_BP_FAT	Translational elongation	2.15E-49	62	3.86E-46
GOTERM_BP_FAT	ribosome biogenesis	5.68E-07	24	1.02E-03
GOTERM_BP_FAT	protein folding	1.48E-06	29	2.67E-03
INTERPRO	IPR017447:CS	1.63E-04	7	2.72E-01
GOTERM_CC_FAT	Intracellular organellelumen	4.76E-05	146	6.88E-02

^a^DAVID Annotation Chart analysis was used to find the most highly enriched group of related terms (with high enrichment score and small FDR) in the Meishan > Yorkshire list of differentially expressed genes. See entire results in Additional file 2: Table S2B.

### Validations by qRT-PCR

**Figure 2:**
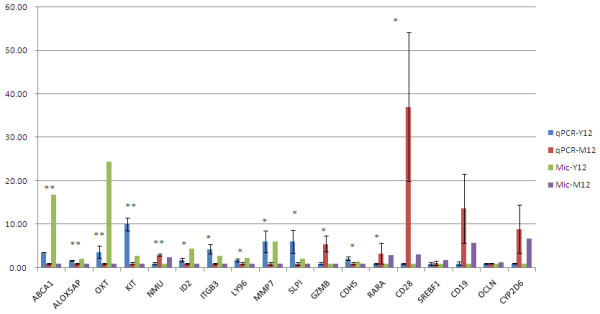
**qRT-PCR validation of genes predicted by Affymetrix GeneChip® analysis to be differentially expressed between Yorkshire and Meishan endometrium on day 12 of gestation.** Blue bars represent qRT-PCR for Yorkshire; red bars represent qRT-PCR for Meishan; green bars represent microarray results for Yorkshire; purple bars represent microarray results for Meishan. Significant differences are indicated with stars:*p < 0.05, **p < 0.01. ABCA1: ATP-binding cassette, sub-family A (ABC1), member 1; ALOX5AP: arachidonate 5-lipoxygenase-activating protein; OXT: oxytocin; KIT: v-kit Hardy-Zuckerman 4 feline sarcoma viral oncogene homolog; NMU: neuromedin U; ID2: inhibitor of DNA binding 2, dominant negative helix-loop-helix protein; ITGB3: integrin, beta 3; LY96: lymphocyte antigen 96; MMP7: matrix metallopeptidase 7; SLPI: secretory leukocyte peptidase inhibitor; GZMB: granzyme B; CDH5: cadherin 5, type 2; RARA: retinoic acid receptor, alpha; CD28: CD28 molecule; SREBF1: sterol regulatory element binding transcription factor 1; CD19: CD19 molecule; OCLN: occludin; CYP2D6: cytochrome P450, family 2, subfamily D, polypeptide 6.

To validate the microarray results, we chose 18 differentially expressed genes identified following the microarray analysis which have also been reported in the literature as relevant to the physiology of implantation. The qRT-PCR results are shown in Figure 2. Of the 18 genes assayed, 14 confirmed statistically significant differential expression in the same direction as the microarray data, and the remaining four genes showed the same direction of expression between microarray and qRT-PCR analyses despite not being statistically different.

### Regulatory network analysis

**Figure 3:**
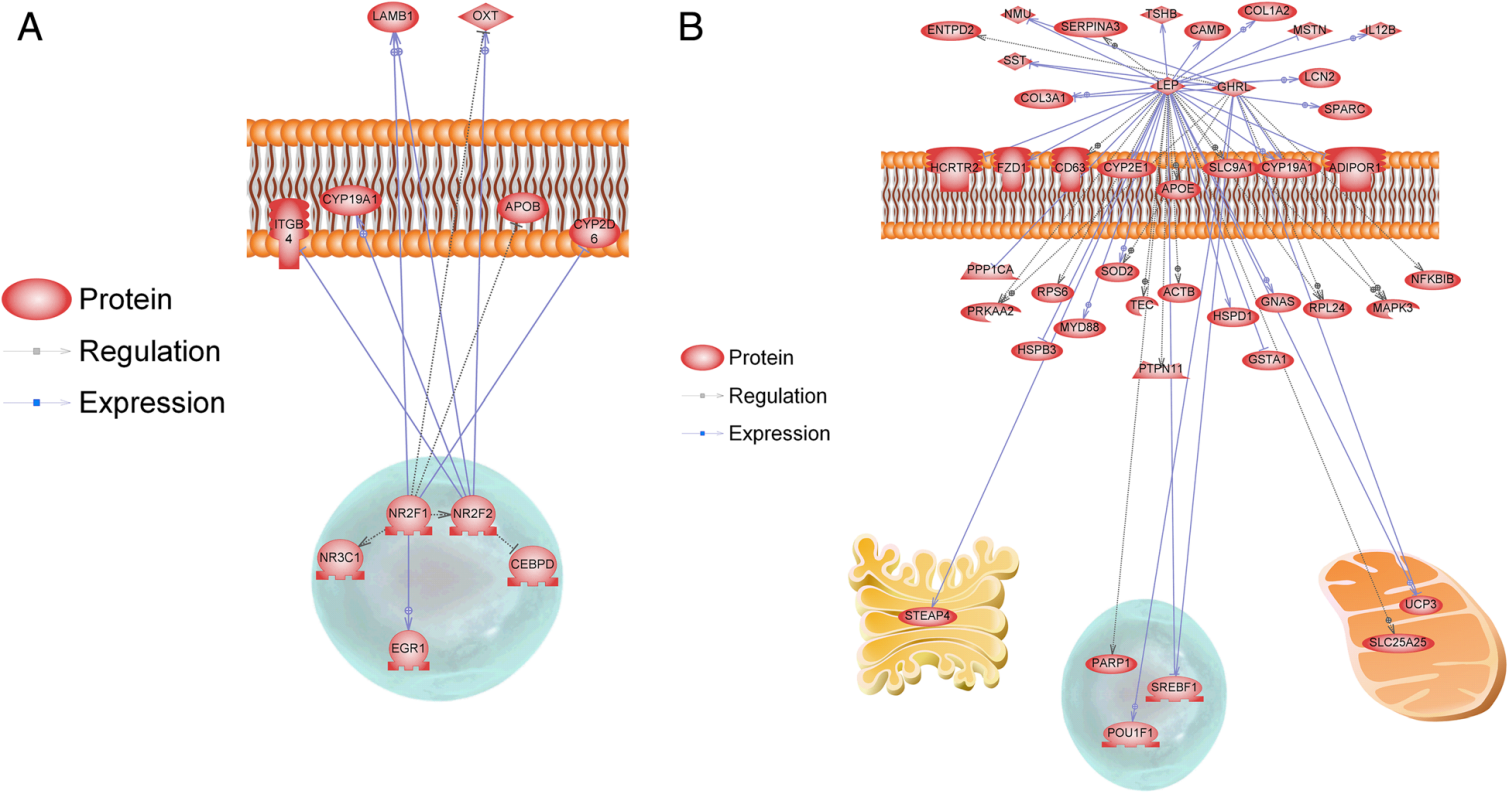
**Sub-Networks of genes regulated by proteins or small molecules created by using Sub-Network Enrichment Analysis (SNEA) software. A**. SNEAresults for RORA (NR2F2) and NR2F1, two enriched proteins using the gene list representing greater abundance for Yorkshire endometrium. **B**. SNEA analyses results for LEP and GHRL, which were enriched using the gene list representing those transcripts more abundantly expressed in the Meishan endometrium on day 12 of gestation compared to Yorkshire.

Identification of small molecules or proteins with a known regulatory link in the biological literature to multiple highly differentially expressed genes is of interest. To identify such sub-networks, a Sub-Network Enrichment Analysis (SNEA) was performed on the gene lists representing those greater in the Yorkshire breed and those greater in the Meishan breed. In all, 37 proteins and 10 small molecules were identified following SNEA with a p-value less than 0.05 (Additional file 3: Table S3). The most significantly enriched protein hub was regulated by RORA, retinoic acid receptor-related orphan receptor alpha (RORA; also known as nuclear receptor subfamily 2, group F, member 2 (NR2F2)) being linked to five genes that were significantly more abundant in endometrium on day 12 from Yorkshire compared to Meishan (Additional file 3: Table S3; Figure 3A). Another retinoic acid receptor, NR2F1, was also highly ranked on the protein list in Yorkshire, due to a link with seven differentially expressed genes (Additional file 3: Table S3; Figure 3A).

Using the list representing DE genes with greater abundance in day 12 endometrium of Meishan, the SNEA identified 59 proteins and 13 small molecules (P < 0.05) as potential regulatory hubs (Additional file 3: Table S3).Of interest, leptin was linked with 38 genes involved in multiple pathways, such as solute carrier family members (SLC25A25, SLC9A1), growth factors (SST, MSTN), and immune system (MYD88, IL12B, CD63); all genes with greater abundance in Meishan endometrium. Also of interest, GHRL was linked with 13 genes more abundantly expressed in Meishan (Additional file 3: Table S3; Figure 3B).

## Discussion

Identifying the endometrial mechanisms and pathways contributing to reproductive success in pigs has long been a research interest [8,32]. The majority of prenatal mortality in pigs occurs before gestation day 20, particularly during the conceptus trophoblastic elongation and attachment to the uterine endometrium, beginning around day 12 of gestation [21]. Establishing the interface between the developing conceptus and the maternal endometrium involves a number of complex signaling networks. It is well known that Meishan pigs farrow 3–5 more piglets than Yorkshire [33]. A study focused on physiological differences between Meishan and Yorkshire embryos showed that *in vitro* cultured Meishan embryos had lower mitotic rate and fewer cells in day 12 conceptuses *in vivo*[34]. Morphological comparison studies revealed that variation of conceptuses diameter was smaller for Meishan pigs, indicating that Meishan conceptuses potentially develop more uniformly between days 8 and 14 of gestation [35,36]. Meishan and Yorkshire embryos transferred to Meishan uterine environment developed slower and secreted less estrogen, compared to being transferred to a Yorkshire recipient, suggesting that the recipient’s genotype was the major factor controlling embryonic growth and estrogen secretion [34]. Comparison of gene expression during physiologically important events across different breeds could enable the identification of novel pathways contributing to reproductive success in pigs. This approach has previously been utilized with the Meishan breed characterizing the ovarian gene expression networks of F2 lberian x Meishan sows [37].

The advantage of microarray analysis is the simultaneous measurement of the expression patterns of large number of genes [38], thus enabling classification of molecular characteristics of phenotypes and generating hypotheses predicting phenotypic outcomes [39]. In the pig, a number of high throughput platforms have been established for global expression profiling analysis [11,15,40-42]. In this study, we conducted a genome-wide transcriptomic analysis on uterine endometrium collected on day 12 of gestation to identify and better understand the molecular basis of differences in conceptus survival between Yorkshire and Meishan pigs. Collectively, we identified the presence of approximately 17,000 expressed transcripts in the uterine endometrium on day 12 of gestation and following statistic alanalysis identified 2656 transcripts with differentially abundance of which 1031 demonstrated 2-fold or greater difference. Following validation of the microarray results with a sub-set of 16 genes assayed with qRT-PCR, we utilized these lists to identify pathways and gene ontology terms associated with each breed by using those gene lists representing expression greater in one breed over another.

### Interpretation of gene annotation analysis: selected pathways of interest

#### Hormone genes and transcriptional activity

Oxytocin (OXT) decreases uterine receptivity in rodents, as rats treated with the OXT antagonist, atosiban, before embryo transfer had improved implantation rates [43,44]. It is of interest that we observed that OXT abundance was greater in Yorkshire compared to Meishan as assessed by both microarray and qRT-PCR. Given that mRNA expression is greater in Yorkshire, it is possible that OXT mediated biological mechanisms may exist that contribute to and/or impact conceptus attachment and interaction with the uterine endometrium.

Prostaglandin production is an important component contributing to the successful establishment of pregnancy in the pig. Expression of prostaglandin endoperoxide synthase 1 (PTGS1) and PTGS2 are substantially increased during implantation [45]. Our microarray results showed that PTGS1 was more abundant in Meishan endometrium (1.7-fold difference, q value = 0.27) whereas PTGS2 was more abundant in Yorkshire endometrium (2.6-fold difference, q value = 0.15), which is consistent with the results in Erhualian endometrium compared to large white [46]. These results indicated that higher PTGS1 may have an important role in embryo survival and attachment in Chinese breeds.

As shown in Additional file 2: Table S2, 157 genes expressed more abundantly in Yorkshire were clustered to the gene ontology (GO) term membrane enclosed lumen, 52 genes to GO term RNA processing, and 40 genes to GO term transcription factor binding pathway following DAVID analysis. Transcription factor transcripts more abundant in Yorkshire, such as HOXA10 and FOXO1, are known to be regulated by steroid hormones, including estrogen, progesterone and testosterone [47-49]. The potential that increased abundance of steroid-hormone dependent factors may be related to the greater quantity of steroid hormone secretion from Yorkshire embryos [34,50].

#### Blood vessel development

Successful apposition and attachment of pig conceptuses followed by the early formation of a placental interface requires a maternal vascular support. We observed that genes detected at a greater abundance in Yorkshire pigs are enriched for GO terms related to blood vessel development. For example, cadherin 5, type 2 vascular endothelium (CDH5) is not only an essential adhesion molecule, but also contributes to angiogenesis which regulates cell proliferation and modulates VEGF receptors [51]. The genes with greater abundance in Meishan, such as PHKA2, PRKAG3, PPP1CA, PYGL, PHKG1, CPS1 were clustered by DAVID analyses into the pathways of glycogen metabolism and glucan metabolism process.

#### Cell adhesion and tight junction genes

Cell-to-cell communication through connexin protein function has been shown in porcine endometrium to be lower than in species with invasive implantation [47]. Thus endometrial cell-to-cell interaction may be involved in limiting trophoblast invasiveness, as porcine implantation is superficial, or in developing specific channels for this implantation type. Proteins such as matrix metalloproteinase (MMP) are endopeptidases that play an important role in the transient invasive property of trophoblastic cells during implantation. Expression of MMP7 was lower in human endometrium in women with repeated implantation failure compared to fertile women, indicating a potential role in human embryonic implantation, an invasive process [52]. In pigs, our qRT-PCR and microarray results show that MMP7 expression is greater in Yorkshire endometrium on day 12 of pregnancy compared to Meishan. Integrin beta 3 (ITGB3) protein has also been detected at porcine embryonic attachment sites on day 24 [47]. ITGB3 has been reported to be involved with signal transduction and formation of cytoplasmic focal adhesions, which was interpreted as supportive for implantation [53]. ITGB3 transcript was in greater abundance in Yorkshire endometrium than Meishan. We also observed other genes expressed more abundantly in Yorkshire compared to Meishan that are annotated as members of tight junction pathways, such as Claudin (CLDN) family members (CLDN1, CLDN5, CLDN8, CLDN10, CLDN11) and tight junction protein 2(TJP2). Tight junction molecules, such as CLDN1, improved invasion activity in cancer cell lines through activation of MT1-MMP-1 and MMP-2 [54], and peak expression of claudin-4 RNA has been temporally associated with the implantation window in humans [55]. These data may suggest that development of membrane channels for specific ions, a major function of claudins, may be important to facilitate successful conceptus attachment in Yorkshire pigs.

#### Immune related pathways

We also investigated the involvement of immune related pathways in DE genes between these breeds based on the importance of immunity in early maternal: fetal interactions. Successful pregnancy requires appropriate spatio-temporal regulation of genes contributing to the immune response during the peri-implantation period. Although reports exist that aborted placentas expressed less Th2 cytokines and greater Th1 cytokines than successful pregnancies [56,57], recent literature indicated that uterine receptivity is actually promoted by a Th1 inflammatory response, whereas the Th2-humoral response facilitated pregnancy maintenance [58]. We observed that genes expressed more abundantly in Meishan endometrium compared to Yorkshire are annotated as functioning in immune response pathways, such as CD28, which may indicate a positive role of CD28 during implantation. CD28 expression has been significant positively correlated with Th1 cytokines expression (IL2 and INFγ) [59]. While reports on mice indicated that suppression of CD28 and other cytokines by cyclosporine A improved pregnancy rate [60], the exact involvement of CD28 in abortion was not clear. In this context, it is interesting that three peptidyl-prolylisomerases (PPIA, PPID and PPIG) are more abundantly expressed in Meishan (Additional file 2: Table S2B). Proteins in the PPI family are known to bind and potentially mediate cyclosporine A function, which in turn has been shown to improve pregnancy rate when administered at the implantation window stage in mice [61]. Thus the PPI proteins may bind to natural cyclosporine analogs impacting the immunological regulation of conceptus attachment.

While not sufficiently enriched at the level of immune annotation clustering, there were also other immune-related genes that are more highly expressed in Yorkshire in the DAVID analysis. Among these, MAPS2 may function by activating complement component factors [62] and alpha-2-macroglobin (A2M) is involved in immune cell migration in pregnancy [63]. Additionally, transferrin (TF) may be involved in removal of allergens from serum [64] and it is expressed differently in the pregnant pig compared the non-pregnant pig [65], indicating the potential role during pregnancy. Higher expression of immunity associated genes such as MAPS2, A2M and TF in Meishan compared to Yorkshire, but not the major cytokines, such as IL2 and IL10, may be suggestive that the difference between these two breeds was not as large as that between females with normal versus aborted pregnancies. However, the SNEA does indicate that genes expressed more abundantly in Meishan are linked to cytokines IL4 and IL6 (Additional file 3: Table S3).

### Regulatory network analysis

RORA (or NR2F2) is a transcription factor belonging to the steroid hormone receptor superfamily [66] and has been demonstrated to be expressed in human endometrium [67]. This transcription factor, as well as NR2F1, has been suggested to play an important role in mediating the cellular response to retinoic acid [68]. RORA is shown to activate hypoxia-inducible factor 1 alpha (HIF-1α) [69] and HIF-1 is an important mediator of estrogen-induced VEGF expression in the uterus [70]. The function of these networks is concordant with the conclusion from our DAVID analysis suggesting greater activation of angiogenesis at this stage in the Yorkshire breed.

Leptin is a small peptide synthesized by white adipose tissue that regulates body weight homeostasis. Leptin transcript abundance was significantly higher in the endometrium of fertile women as compared to women with recurrent implantation failure [71] and thus higher Meishan expression of Leptin in our study is consistent with the prolificacy seen in this breed. Ghrelin is thought as a novel paracrine/autocrine factor expressed in endometrium and placenta that is involved in crosstalk between endometrium and embryo during embryo implantation [72], which may indicate that such intra-tissue communication is critical for implantation success.

## Conclusions

Conceptus and fetal loss during early pregnancy establishment can be a critical determinant of litter size. However, identification of molecular pathways that may contribute to successful pregnancy establishment and the formation of a fetal: maternal interface has not yet been investigated in breeds with established differences in litter size performance. In this study, the Affymetrix GeneChip® was used to compare gene expression profiles and identify associated pathways which may be differentially regulated in the uterine endometrium of Meishan and Yorkshire gilts during day 12 of gestation. The results have described the molecular portraits of uterine endometrium during the opening of the implantation window revealing differences between the Meishan and Yorkshire breeds. Our results revealed several classifications of genes participating in regulation of endometrium function during early pregnancy. Future analysis will require an exhaustive look into the protein repertoire of the uterine endometrium of the two breeds to strengthen our understanding of the relationship between the transcriptome and proteome in the uterine endometrium.

## Methods

### Animals

All animal use was performed under Iowa State University Committee on Animal Care approved protocols. Heat-check positive Yorkshire and Meishan gilts were bred twice to boars of the respective breed, 24 h apart, via natural service at their second or third estrus. Reproductive tracts were recovered immediately after slaughter at ISU using IACUC approved protocols. The gestation date was calculated from the first mating day. Endometrium was collected from 10 cm sample near junction with other uterine horn at gestation day 12. Following removal of the reproductive tract, the uterus was opened by cutting down the anti-mesometrial border and uterine endometrium was collected from all areas of the lumen and snap-frozen in liquid nitrogen. If no elongated conceptuses were observed in a bred female, tissue samples from that female were not used in the study. Uterine endometrial samples from a total of four Yorkshire and four Meishan gilts were chosen using these criteria.

### RNA preparation

Total RNA was isolated from 200 mg of tissue using the QIAGEN RNeasy kit (Cat no.714104). The DNA was removed by DNase I digestion and RNeasy mini elute kit cleanup as recommended by QIAGEN. PCR assay without reverse transcription was used to confirm that the RNA samples were absent for genomic DNA. The quantity and quality of the RNA were determined using Agilent 2100 Bioanalyzer(Agilent Technologies, Santa Clara, CA) and Nanodrop 2000 (Thermo Scientific, Wilmington, DE). RNA samples with RIN number lower than 7 or yield less than 3 μg was identified as low quality and excluded from the experiment.

### Microarray hybridization and statistical analyses

The porcine genome microarrays were purchased from Affymetrix (Cat. no. 900623; Santa Clara, CA). The Affymetrix GeneChip® Porcine Genome Array contains 23,937 probesets which represents 20,201 genes and 190 controls. The sequence information for this array was selected from public data sources including UniGene Build 28, GenBank® mRNAs. The RNA labeling, porcine gene chip hybridization, washing and signal detection were done at the GeneChip Facility, Iowa State University, Ames, IA according to the manufacturer’s instructions.

Data from .cel files were converted to gene signal files by GCOS1.2. Natural log was taken for all the raw gene signals for normalization. The SAS ANOVA analysis was used to identify differential gene expression (SAS Institute, Cary, NC). A mixed model including random effects for slide and animal was fit to the expression data for each gene using GLM procedure in SAS. The p-values were obtained for the Meishan and Yorkshire comparison. The p-values for each test were converted to q-values for false discovery rate estimation [73].

### Real-time RT-PCR

RNA samples from the eight animals which were used for the Affymetrix experiment were analyzed by quantitative reverse-transcription PCR. The reverse transcription was performed using SuperScript II Reverse Transcriptase and Oligo(dT) primer according to the manufacturer’s instructions (Invitrogen, Carlsbad, CA). All reverse transcription reactions were run along with no-template controls. The no-template controls gave non-detectable signals in all samples, confirming the high specificity of the assays. Real-time PCR was performed using Stratagene Brilliant Kit (La Jolla,CA). The sequences for probes and primers have been previously published [74]. All reactions were run in duplicate and data were normalized using the house keeping gene RPL32. The significance level was set to 0.05.

### Differentially expressed gene list analysis

#### Clustering analysis

An unsupervised learning procedure in Gene Cluster 2 software was used for the cluster analysis and used normalized LSMean values of differentially expressed genes. Hierarchical cluster analysis was done by using Cluster and TreeView software (http://rana.lbl.gov/EisenSoftware.htm). The p-value cut off used for differential expressed genes in the cluster analysis is p < 0.005 (q < 0.05).

#### Pathway studio analysis of common pathways represented by DE genes in a group

Pathway Studio 9.0 (Ariadne Genomics, Rockville, MD, US). This software was used to find common pathways determined by differentially expressed genes and subsequently gain a better insight in Yorkshire versus Meishan day 12 endometrium expression profiles. Pathway Studio is a text-mining tool that scans manuscripts and abstracts from multiple biomedical web resources to establish known relationships. We used the differentially expressed list at q < 0.01 and performed a sub-network enrichment analysis (SNEA) on the up- and down-regulated gene lists. Our analyses focused on proteins and small molecules.

### Availability of the supporting data

All Affymetrix data has been submitted to GEO database with accession number GSE46332 (http://www.ncbi.nlm.nih.gov/geo/query/acc.cgi?token=kpifaowqnjczdut&acc=GSE46332).

## Abbreviations

MAS: Marker-assisted selection; DE: Differentially expressed; M12: Meishan day 12; Y12: Yorkshire day 12 endometrium; RORA: Retinoic acid receptor-related orphan receptor alpha; NR2F2: Nuclear receptor subfamily 2, group F, member 2; LEP: Leptin; GHRL: Ghrelin; OXT: Oxytocin; PTGS1: Prostaglandin endoperoxide synthase 1; VEGF: Vascular endothelial growth factor; CDH5: Cadherin 5, type 2 vascular endothelium; MMP: Matrix metalloproteinase; ITGB3: Integrin beta 3; CLDN: Claudin; TJP2: Tight junction protein 2; PPI: Peptidyl-prolylisomerases; EPHX2: Epoxide hydrolase; TF: Transferrin; GO: Gene ontology; SNEA: Sub-network enrichment analysis.

## Competing interests

The authors have declared no competing interests.

## Authors’ contributions

TG performed the DAVID analyses, wrote the majority of the first draft of the manuscript and edited the final version. MJZ wrote part of the draft and assisted TG in making heatmap for microarray differentiation genes. MS ran the Pathway Studio analysis and provided interpretation and text for that section. LQ and DN performed the statistical analysis of the microarray data. DK and JKL performed the Q-PCR analysis. SHZ, JWR and CKT collected tissues and assisted in interpretation of the data and writing the manuscript. All authors read and approved the final manuscript.

## Supplementary Material

Additional file 1: Table S1 Microarray results. Click here for file

Additional file 2: Table S2 A and B: GO analysis pathways. Click here for file

Additional file 3: Table S3 SNEA analyses results. Click here for file
